# Agreement between Fathers’ and Mothers’ Reported Stimulation and Associations with Observed Responsive Parenting in Pakistan

**DOI:** 10.3390/children6100114

**Published:** 2019-10-15

**Authors:** Joshua Jeong, Saima Siyal, Aisha K. Yousafzai

**Affiliations:** 1 Department of Global Health and Population, Harvard T.H. Chan School of Public Health, Boston, MA 02115, USA; ayousafzai@hsph.harvard.edu; 2 Department of Pediatrics and Child Health, Aga Khan University, Karachi 74800, Pakistan; saima.siyal@aku.edu

**Keywords:** parenting, stimulation, responsiveness, parent–child interaction, fathers, early childhood, Pakistan

## Abstract

Parental stimulation and responsiveness are associated with improved early child development outcomes. However, the majority of studies have relied on maternal-reported measures of only mothers’ parenting practices. The purpose of this study was to assess the agreement between fathers’ and mothers’ reports of their own and their partner’s engagement in stimulation and assess the degree to which parents’ reported stimulation correlated with their observed responsive caregiving behaviors. Data were collected from 33 couples (33 fathers and 32 mothers) who had a child under 5 years of age in rural Pakistan. Paternal and maternal stimulation were measured based on reports of their own and their partner’s practices in play and learning activities with the child. Paternal and maternal responsiveness were observed in a subsample of 18 families. Moderate agreement was found between paternal and maternal reports of their own and their partner’s practices. Moderate associations were also found between self-reported measures of stimulation and observed responsive caregiving for both fathers and mothers. The strengths of agreement and associations were greater among couples who had higher quality coparenting relationships. Findings highlight the feasibility, reliability, and promise of assessing fathers’ parenting in a low-resource setting, using similar methods as for mothers’ parenting, to triangulate measures between reported and observed parenting and gain a deeper understanding of fathers’ and mothers’ unique caregiving contributions.

## 1. Introduction

It has been estimated that 250 million or 43% of children under 5 years are at risk of not attaining their developmental potential [1,2]. Population-based multi-country studies have shown that mothers are substantially less engaged in stimulation (e.g., reading books, playing, naming) with young children in low- and middle-income countries (LMICs), compared to mothers in high-income countries [3]. The critical role of parents in providing early learning opportunities and engaging sensitively and responsively have been emphasized as two key components of the Nurturing Care Framework to promote early child development (ECD) globally [4]. Consequently, parenting interventions that aim to encourage parents’ shared play and communication with their children, build parenting skills, and enhance the quality of parent–child interactions have been increasingly implemented in LMICs [1]. Such parenting interventions have shown medium-sized effects for improving young children’s cognitive and language development outcomes [5].

Despite the growing implementation of interventions, there has been slower progress with regards to the measurement of parenting in LMICs. The majority of studies to date have relied on caregiver-reported measures of parenting, such as the most commonly used Family Care Indicators (FCI) [6,7,8]. The FCI is a 17-item, caregiver-reported measure about whether or not any adult in the home engaged in stimulation activities with the child in the past 3 days (e.g., reading, singing, naming things) and varieties and sources of play materials [9,10]. Although this measure is quick to administer and has shown predictive validity with child development outcomes [9], it is limited to self-reports of stimulation practices, and does not capture frequency, quality, or more specific dimensions of parenting behaviors (e.g., sensitivity and responsiveness).

Observational measures are regarded as the gold standard for assessing parenting [11], and aspects like parental sensitivity and responsiveness are more nuanced and predictive of ECD outcomes than reported stimulation practices [12,13]. Of note, a few studies have used observational measures to assess these specific parenting behaviors in LMICs. For instance, Aboud and Akhter [14] used a picture book activity to observe maternal directive and responsive speech with children during the first two years of life in rural Bangladesh. Rasheed and Yousafzai [15] developed the Observation of Mother–Child Interactions (OMCI) tool, a similar picture book activity with a standardized scoring protocol, to capture maternal responsive caregiving and children’s behaviors during a 5-min picture book activity. This tool has demonstrated good reliability, construct validity, and predictive validity upon use with a large sample and broad range of children aged 12–48 months in rural Pakistan [16,17] and is being increasingly used in other LMIC contexts [18,19].

However, the majority of studies measuring parenting of young children have focused exclusively on mothers. Of notable exception, a few studies have shown that fathers’ sensitivity and responsive stimulation independently influence children’s cognitive, language, and socioemotional development outcomes, above and beyond mothers’ parenting [20]. Moreover, family systems theory underscores how parent–child dyadic relationships are embedded within broader family interactions among children and multiple caregivers and also shaped by the coparenting relationship between couples [21]. Despite this empirical and theoretical literature, fathers’ parenting behaviors have been largely overlooked and unmeasured in LMICs. A few recent studies in LMICs have made advancements in this literature by adapting the original wording of the FCI and asking mothers to report about not only their own stimulation, but also their partner’s (i.e., paternal) stimulation [22,23]. Such efforts have revealed important new findings regarding the comparative roles of mothers and fathers. For example, using data from the Multiple Indicator Cluster Survey, which asks primary caregivers about maternal and paternal stimulation, Jeong et al. [22] found that fathers in LMICs were significantly less engaged in stimulation with their preschool-aged children than mothers. 

Although mothers’ reports on fathers’ parenting provides a more expansive perspective and important indication of other caregivers’ unique contributions, these data are also potentially subject to an additional source of reporting bias, as it is not entirely clear whether mothers are accurate reporters of fathers’ practices [24,25,26]. A recent meta-analysis revealed a weak, yet significant, association between broadly any parent-reported and observed parenting measure [27]. Determining the strength of the association between reported and observed parenting measures, and whether there are any factors that explain the strength of this association, would shed light on the construct validity between these measures for assessing parenting and aid in the decision-making considerations between measurement options.

The majority of existing literature in this field is based on data from the United States and other high-income countries. Yet, it has been well-documented that parenting practices are much lower in LMICs than HICs due to a variety of greater reasons, including poverty, low parental education, family stress, and other social determinants [28]. Additionally, many societies across LMICs—including Pakistan—have strong patriarchal values and norms [29,30]. For example in Pakistan, most mothers are unemployed, expected to remain at home doing house chores for the extended family, excluded from decision-making, and subservient to their male partners [31]. Although some parenting roles, such as early instruction and looking at the health of the child, are more common among both mothers and fathers, many other roles and responsibilities are clearly delineated as maternal versus paternal roles in rural Pakistan [32]. Considering some of these cultural differences, an investigation of maternal and paternal parenting of young children is critically needed from settings such as Pakistan and elsewhere around the world.

In this study, we use primary data collected from fathers and mothers of young children under 5 in rural Pakistan. This study aimed to: (1) describe fathers’ and mothers’ stimulation and responsive caregiving; (2) assess the agreement between fathers’ and mothers’ reports of their own and their partner’s parenting practices; (3) assess the degree to which fathers’ and mothers’ reported practices were correlated with observed responsive caregiving behaviors; and (4) explore whether these relations differed by the quality of the couple’s coparenting relationship.

## 2. Methods

### 2.1. Participants and Procedures

Data for this study come from a primary study that aimed to understand the lived experiences, perceptions, and personal meanings that parents ascribed to fathers’ and mothers’ caregiving roles for children under five years of age in Naushehro Feroze district, in Sindh province, Pakistan [32]. Naushehro Feroze is an impoverished rural community, where 32% of households are food insecure, 29% of fathers and 68% of mothers are illiterate, 7% of children under five years of age have three or more books at home, and 18% of preschoolers attend an early education program [33]. Two Pakistani research assistants recruited households from January to March 2017 using household lists generated by the local community health worker program and the field research team. Households with children under aged five years were randomly selected from this list, and the adult male of the household was contacted via phone or in-person to determine whether he met the inclusion criterion of being the biological father to a child aged less than five years old, and was interested in participating. All households selected for contact agreed to participate. A stratified sampling strategy was used to achieve an equal distribution of households based on three characteristics: paternal education (less than secondary schooling and secondary school or higher), child gender (male and female), and child age (0–2 and 3–5 years).

Data collection typically occurred soon after enrollment (i.e., the following day). Two research assistants (one male and one female) scheduled and made home visits, in pairs, to the households. Upon visiting the home for the scheduled visit with the father, the child’s mother was also approached to determine her interest in also participating in the study. In one household a mother declined to participate. After completing the informed consent process, the research assistant administered a brief survey and then conducted an in-depth interview with the parent. The male research assistant worked with the father, while the female research worked with the mother in separate private locations. In total, 33 families (33 fathers and 32 mothers of 33 children) participated in the survey and in-depth interviews.

Finally, in addition to administering the survey and in-depth interviews, the research assistants asked parents whether they were willing to participate in a 5-min shared book reading activity with the child that would be video-recorded. Additional consent was gathered for this activity. Of those 33 families in the parent study, 18 families participated in the observed shared book reading activity (18 mother–child videos and 18 father–child videos). The main reason for refusal was that the father did not grant permission for his family to be video-recorded. However, there were no differences in parent or household characteristics between those families who participated versus did not participate in the observed book reading activity. Ethics approval for this study was obtained from the institutional review board at the Harvard T.H. Chan School of Public Health (IRB16–1116) and the ethical review committee of the Aga Khan University (4428-Ped-ERC-16). All participating respondents gave informed written or verbal consent.

### 2.2. Measures

Parents’ stimulation was measured using an adapted version of the FCI tool [10]. Mothers and fathers completed the survey, with each parent reporting on the frequency of both their own and their partner’s engagement in seven play and learning activities with the child in the past week: reading books, singing songs, taking the child out of the home, playing, naming things, drawing things, or telling stories. Each item was reported on a four-point scale: 0 (never), 1 (once or twice a week), 2 (multiple times a week), and 3 (every day or nearly every day). Total scores were generated, with higher scores indicating more engagement in stimulation.

Parents’ responsive caregiving was observed during a 5-min dyadic interaction. The parent was instructed to interact with the child as they normally would using a picture book that was provided by the study team. These interactions were video recorded by the research assistants and later scored by two independent coders using an adapted version of the Observation of Mother and Child Interaction (OMCI) protocol [15]. This specific tool has been previously used to capture reliable and valid measurements of parental sensitivity and responsivity [16,17,18,19]. In addition, prior studies have shown that observational methods for assessing parent–child interactions are feasible and acceptable for use with parents and children in low-income, non-Western cultural contexts [34]. Two coders rated the videos for 19 different parental or child behaviors including: parental positive and negative affect; positive and negative touch; positive and negative verbal statements; sensitivity; scaffolding; pointing and asking questions; answering questions; and helping the child maintain interest (see Rasheed & Yousafzai, 2015 for more details). Coders rated each of the 19 behaviors on a five-point scale: 0 (never), 1 (rarely), 2 (sometimes), 3 (often), and 4 (always). Coders were trained and reached sufficient inter-rater reliability (Kappa > 0.80) using videos of parent–child interactions from another study before beginning the coding. The inter-rater reliability between the two coders for the final sample of videos was Kappa = 0.94. Parental behavior items were summed together (with negative items reverse coded) for a total score (plausible range: 0–95), such that higher scores reflected more positive and responsive caregiving.

Coparenting relationship quality was coded from the in-depth interview transcripts. These data largely emerged in response to the following two questions in the semi-structured topic guide: (1) What parenting activities are strictly the mothers’ role? fathers’ role? And what parenting activities are both mothers and fathers responsible for? (2) How, if at all, do you support your partner in raising your child? Qualitative data were analyzed using thematic content analysis by two independent coders using an iteratively developed codebook and in NVivo. Details about these coding procedures have been previously published [32]. In this study, we reviewed the supporting quotes associated with the ‘coparenting relationship quality’ code. Considering the evidence from both the mothers’ and fathers’ transcripts, we categorized couples as having described either relatively high or low coparenting. For example, the following is a quote from a father that suggested high coparenting: “We do a lot of things together to keep the child happy. If my wife is not free, then I pick up [child’s name]. And if I forget something, then my wife gets it when she goes to the market. We don’t do like the chores only a wife will do. We both do everything together. 50% I play a role, and 50% my wife.” The following is a quote from a father that suggested low coparenting: “I don’t feed or play with him because he [child] has a mother and she cares for him without me… The father cannot care for the child as much as the mother”. We categorized the few couples who did not mention coparenting in their relationship as low.

### 2.3. Analysis

First, we conducted two paired-sample *t*-tests to determine if there were significant differences in the mean levels of parenting between fathers’ and mothers’ self-reported stimulation practices and observed responsive caregiving behaviors. Second, focusing on the reported stimulation practices, we conducted two paired-sample *t*-tests to assess whether parents differed in their reports about a given parent’s practices (i.e., comparing fathers’ self-reported practices with mothers’ reports of their husbands’ practices). We further calculated intraclass correlations (ICC) to assess the degree of agreement in parental stimulation between a parent’s self-report and their partner’s report of their practices. Third, we calculated a series of Pearson correlations to explore the degree to which parents’ self-reported stimulation correlated with their own observed responsive caregiving behaviors. We considered statistical significance up to the *p* < 0.10 level, given the small sample size [35]. Finally, we coded transcripts from in-depth interviews with fathers and mothers to determine quality of the coparenting relationship. We qualitatively assigned the sample into two groups—high versus low coparenting—and explored correlations between maternal and paternal reports of a given parent’s stimulation and between self-reported stimulation and observed responsive caregiving behaviors for the two sub-groups. Quantitative analyses were conducted in Stata 15, and qualitative analyses were conducted in NVivo 12.

## 3. Results

The sample comprised of 65 parents, representing 33 families (33 fathers and 32 mothers of 33 children). Approximately half of fathers had less than primary school (46.6%) versus secondary school or higher (53.3%). The majority of mothers (59.4%) had no education. Fathers most commonly worked in agriculture (33.3%) or some casual labor job (33.3%; e.g., miller, carpenter, driver). Six fathers lived apart from their children and family for extended periods of time due to their migratory employment. Nearly half (48.5%) the children of sampled fathers were female and slightly greater than half were of the younger age-group of children aged 0–2 years (54.5%). The majority were joint household structures (79%), in which families resided together with other extended family members.

First, we were compared self-reported practices and observed behaviors between fathers and mothers. Means, standard deviations, and internal consistencies for fathers’ and mothers’ parenting measures are presented in Table 1. Based on the results of two paired-sample *t*-tests, there were no significant differences between fathers’ and mothers’ mean self-reported levels of stimulation or observed responsive behaviors.

Second, we assessed the degree to which fathers and mothers agreed about the reports of their own and their partner’s practices. On average, fathers reported their wives as being more engaged in stimulation than mothers reported about themselves, *t*(32) = 2.83, *p* < 0.01. Mothers’ reports of their husbands’ practices aligned with and did not differ from how fathers described themselves. There was a fair degree of agreement between paternal and maternal reports of fathers’ behaviors (ICC = 0.53) and marginally weaker agreement between paternal and maternal reports of mothers’ behaviors (ICC = 0.43).

Third, we determined the correlations between fathers’ and mothers’ reported practices and observed behaviors. A correlation matrix of all primary study variables is presented in Table 2. There were moderate positive relationships between parent’s self-reported stimulation practices and their own responsive behaviors (*r* = 0.43, *p* < 0.10 for fathers; *r* = 0.59, *p* < 0.01 for mothers).

Lastly, we compared agreements in stimulation as reported between couples, and correlations between a parent’s own reported stimulation practices and observed responsive behaviors, by coparenting relationship quality subgroups (high versus low). In total, 16 out of 33 couples (48.5%) were categorized as high coparenting. We found that agreement between paternal and maternal reports of fathers’ stimulation was substantially stronger among couples who had high quality coparenting relationships (ICC = 0.70) compared to couples who had low quality coparenting relationships (ICC = 0.15). Similarly, the agreement between paternal and maternal reports of mothers’ stimulation was substantially stronger among couples who had high quality coparenting relationships (ICC = 0.76) compared to couples who had low quality coparenting relationships (ICC = 0.16). The correlations between a parent’s self-reported stimulation practices and observed responsiveness were stronger among couples with high quality coparenting relationships (*r* = 0.86 for mothers; *r* = 0.64 fathers) than couples with low quality coparenting relationships (*r* = 0.61 for mothers; *r* = 0.39 for fathers).

## 4. Discussion

Parenting is often measured using self-reports and primarily among mothers exclusively. The purpose of this study was to examine agreement in self-reported parenting styles of fathers and mothers of children less than five years old, assess the degree to which fathers’ and mothers’ reported practices were correlated with observed parental behaviors, and explore whether these relations differed by the quality of the couple’s coparenting relationship. This study revealed moderate agreement between paternal and maternal reports of their own and their partner’s practices and a positive medium-sized correlation between parenting measures based on self-report and observations.

Overall, we found that fathers and mothers reported similar levels of engagement in stimulation and showed similar levels of sensitivity and responsiveness in their interactions with children. Our findings contribute to the relatively mixed body of evidence, predominantly from high-income countries, regarding the quantitative similarities and differences between maternal and paternal parenting. Some prior studies have similarly determined no significant differences between mothers’ and fathers’ reported practices [24] or observed parenting behaviors with young children [36]. On the other hand, several studies have demonstrated that fathers over-report engaging in parenting activities than mothers [25,37], whereas mothers receive significantly higher ratings on observed parenting dimensions like sensitivity and emotional availability [20,38].

We found moderate agreement between paternal and maternal reports of their own and their partner’s practices. There was a slightly weaker agreement between paternal and maternal reports of mothers’ practices than fathers’ practices, as fathers on average were more likely to report their wives as being more engaged in stimulation than mothers reported about themselves. Our findings showing the considerable variability and moderate agreement in fathers’ and mothers’ reports of parenting a young child less than five years old are consistent with prior research [24,39,40]. For example, using data from low-income families living in Boston, Chicago, and San Antonio in the United States, Coley and Morris [24] found that the average level of agreement in reports of paternal parenting during early childhood was 61% and the correlation between paternal and maternal reports was 0.69. In our sample, parents rated themselves and their partners generally quite similarly, indicating a low level of discordance in perceptions of parenting styles.

We also found that self-reported measures of stimulation practices were positively correlated with observed sensitive and responsive behaviors among both fathers and mothers. A recent meta-analysis demonstrated that there is a small but positive overall association between parent-reported and observed measures of parenting [27]. Of note, each of the 36 articles included in this meta-analysis were from high-income countries and represented samples that were nearly entirely all mothers. Our results extend this body of literature to date by demonstrating a moderate correlation between parent-reported and observed parenting in an under-studied sample in rural Pakistan and particularly revealing that the association between parent-reported and observed parenting is also supported not only among mothers but also fathers. In fact, this is one of the first known studies to observe father–child interactions in a LMIC context. This finding suggests that measuring the frequency of parents’ engagement in developmentally stimulating activities—such as reading, story-telling, singing, or playing—may provide an initial first-step indication of parents’ responsive behaviors [41]. 

However, at the same time, results underscore that parental stimulation and responsiveness are quite distinct, such that other factors uniquely explain variation in parental responsiveness above and beyond stimulation. For instance, prior research in South Asia has shown that maternal knowledge of ECD, maternal depression, household wealth, and child age predict maternal responsive behaviors [15,19,42]. Future research should collect a variety of behavioral, psychosocial, and demographic characteristics to decompose the relative determinants of parental responsiveness. Overall, the results of our study suggest that encouraging increased frequency of play and communication does not directly improve quality of interactions. Therefore, parenting programs should also incorporate additional strategies, such as directly coaching and supporting parents to interactively practice and build skills such as sensitivity, responsivity, emotional warmth, and scaffolding.

Finally, we found that these relations—both the degree of agreement between fathers’ and mothers’ reports of a given parent’s practices and also the strength of the correlation between the reported and observed parenting measures—differed by the quality of the couple’s coparenting relationship. More specifically, these relations were markedly stronger among couples who described high coparenting relationships. Our findings are consistent with prior related studies that have shown that poorer couples’ relationship quality [25] and couples’ conflict [24] predict higher levels of discrepancy between fathers’ and mothers’ reported parenting practices. A possible explanation for this could be that among couples with poorer relationships or high conflict, parents downplay the contributions of their partners or over-report their own relative parenting roles [24]. Several studies have suggested other child-, parent-, and family-level factors that predict the degree of agreement between paternal and maternal reports and correlation between reported and observed parenting behaviors, including child age, paternal residential status, paternal age, and maternal education [25,41]. Future research should explore whether these and other sociodemographic factors further explain the shared variance between raters and measures, as this information can be used to both methodologically and conceptually improve survey design and data collection consideration for assessing maternal and paternal parenting.

There are several limitations to this study. First, the sample size was relatively small (65 parents reporting on 33 children). Approximately half of parents agreed to also be video recorded (18 families), yielding a low response rate. The small sample size limits the power of our analyses, and the selection bias limits the generalizability of our findings to broader populations. Our results should certainly be replicated with larger samples. Second, the measures were somewhat limited. The measure for parenting practices specifically focused on reported frequency of engagement in seven stimulation activities. Yet, fathers’ parenting encompasses other aspects beyond stimulation and responsiveness (e.g., warmth, control, financial support) [43]. The measure for observed responsive behaviors focused on a structured 5-min play session between the parent and child using a picture book. Parents’ observed behaviors during this single context may not accurately reflect their typical parenting behaviors. High and low coparenting relationship quality was determined based on in-depth interviews. Although these data were coded and compared between two independent analysts, they are subject to the level of detail shared by the respondents. Further measurement research is needed to inform the adaptation and development of standardized co-parenting surveys that are reliable and valid for use in the Pakistani context. Finally, it is also important to highlight that the findings may not be generalizable using other reported or observed measures of parenting and in other populations around the world.

## 5. Conclusions

Our findings show that the frequency of parental engagement in stimulation activities (e.g., reading books, naming things) is moderately related to quality of observed parenting (e.g., sensitivity, responsiveness, emotional warmth). This suggests that parenting programs that principally aim to increase parental stimulation may not be sufficient for enhancing quality of parent–child interactions. Previous trials have demonstrated how other intervention components and strategies, such as home-visiting programs during which coaches provide feedback to parents regarding how to sensitively and appropriately respond to their child’s cues, are effective in enhancing parental sensitivity and responsiveness and improving ECD outcomes [44,45]. However, our finding also revealed that the associations between fathers’ and mothers’ reported and observed parenting were moderate—suggesting that these parenting constructs (i.e., practices and observed behaviors) are also distinct and represent different functions and skills [46]. Together, these findings highlight the importance of using measurement tools that are specific for the constructs of interest. The majority of parenting intervention evaluations in LMICs have used maternal-reported measures of caregiving practices; very few studies have used observational measures of parent–child interactions or given attention to fathers. Considering the Nurturing Care Framework and specifically the unique importance of responsive caregiving [1], the OMCI is one observational measure that can be used to capture this key aspect of mothers’ and fathers’ care for child development and complement a measure of parent-reported practices. Our study is one of the first to show that the OMCI is feasible for use with specifically fathers in a LMIC context.

Overall, our study highlights that both mothers’ and fathers’ parenting should be specifically disentangled and multiple informants should be included when possible, considering how multiple caregivers commonly comprise the family system. In fact, our findings suggest that including one parent’s perspective may provide an incomplete or potentially biased perspective of a parent’s contributions, especially when there are poor relationships between couples. In conclusion, the use of multiple informants and multiple measures of fathers’ and mothers’ parenting—including self-reports of parenting, reports of spouse’s parenting, and independent observational measures of both parents’ parenting practices—will provide new insights into the complex processes by which parents care for their children and yield more ecologically valid measurements of caregiving that represent the roles of not only mothers but also fathers. Future research should include fathers and investigate how these different dimensions of maternal and paternal parenting relate to early child development outcomes in low-resource settings.

## Figures and Tables

**Table 1:** Descriptive statistics for reported measures of paternal and maternal stimulation and observed measures of paternal and maternal responsiveness.

Statistic	Father Stimulation		Mother Stimulation		Observed Father Responsiveness	Observed Mother Responsiveness
	Reported by Father	Reported by Mother	Reported by Father	Reported by Mother		
Mean	1.50	1.52	1.69	1.38	53.67	52.77
SD	0.83	0.71	0.72	0.73	10.01	11.26
Range	0–3	0.57–3	0.43–2.86	0.14–3	36–66	34–70
Alpha	0.81	0.75	0.73	0.70	0.89	0.93

**Table 2:** Correlation matrix of reported measures of paternal and maternal stimulation and observed measures of paternal and maternal responsiveness.

Variable	Father Stimulation (Father Report)	Father Stimulation (Mother Report)	Mother Stimulation (Father Report)	Mother Stimulation (Mother Report)	Father Responsiveness (Observed)	Mother Responsiveness (Observed)
Father stimulation (father report)	1.00					
Father stimulation (mother report)	0.34 *	1.00				
Mother stimulation (father report)	0.52 ***	0.31 *	1.00			
Mother stimulation (mother report)	0.40 **	0.49 ***	0.62 ***	1.00		
Father responsiveness (observed)	0.43 *	0.61 ***	0.11	0.26	1.00	
Mother responsiveness (observed)	0.54 *	0.51 *	0.15	0.59 **	0.69 ***	1.00

* *p* < 0.10, ** *p* < 0.05, *** *p* < 0.01.
